# *Photobacterium damselae* subsp. *damselae* at the animal-human interface: ecology, pathogenicity, and genomic gaps in a changing climate

**DOI:** 10.3389/fmicb.2026.1901411

**Published:** 2026-08-12

**Authors:** Marzia Sapio, Matteo Mellace, Xosé M. Matanza, Rosa Luisa Ambrosio, Antonino Pace, Bruno Tilocca, Adriano Minichino, Alessandro Fioretti, Ludovico Dipineto, Luca Borrelli

**Affiliations:** 1 Department of Veterinary Medicine and Animal Production, University of Naples Federico II, Naples, Italy; 2 Department of Health Sciences, University Magna Græcia of Catanzaro, Catanzaro, Italy; 3 Department of Life Sciences and Centre for Bacterial Resistance Biology, Imperial College London, London, United Kingdom; 4 Department of Veterinary Medicine, University of Sassari, Sassari, Italy

**Keywords:** climate change, cross-species transmission, marine pathogen, One Health, *Photobacterium damselae* subsp. *damselae*, potential emerging pathogen, *Vibrionaceae*, wildlife

## Abstract

*Photobacterium damselae* subsp. *damselae* (*Pdd*) is a Gram-negative bacillus with zoonotic potential and wide global distribution. Found primarily in marine ecosystems, *Pdd* has been reported in diverse hosts, including fish, mollusks, crustaceans, marine mammals, sea turtles, birds, and humans. Infection dynamics are influenced by host factors such as environmental stress and pre-existing conditions, while clinical signs are frequently non-specific, making misdiagnosis likely. Its virulence involves iron acquisition systems, a polysaccharide capsule, hemolysins, and other cytotoxic enzymes, some of which are plasmid-encoded and whose expression is influenced by environmental conditions such as water temperature and salinity. This narrative review reports the current knowledge on *Pdd*’s pathogenic mechanisms, ecological traits, diagnostic approaches, and histopathological features of infections reported in various hosts. It also addresses antimicrobial resistance patterns and alternative prevention and control strategies, available genomic data, and future bioinformatics perspectives. As a conceptual framework, we propose climate change as a dual driver that compromises host defenses and may promote pathogen persistence and virulence, thereby potentially facilitating opportunistic infections and cross-species transmission. By highlighting knowledge gaps, this review aims to inform future surveillance efforts and interdisciplinary research on the ecological and epidemiological dynamics of *Pdd* in wildlife, livestock, domestic animals, and humans.

## Introduction

The genus *Photobacterium* is classified within the family *Vibrionaceae*. Its members are Gram-negative coccobacilli, or occasionally rods, typically ranging from 0.8 to 1.3 μm in diameter and 1.8–2.4 μm in length (Moi et al., 2017).

To date, this genus contains 40 species that are officially recognized by the International Code of Nomenclature of Prokaryotes (ICNP) (LPSN, 2026). Most of these species have been isolated from marine environments and organisms worldwide, establishing mutualistic, commensal, or parasitic interactions (Labella et al., 2017).

*Photobacterium damselae* is divided into two subspecies, *Photobacterium damselae* subsp. *damselae* (*Pdd*) and *Photobacterium damselae* subsp. *piscicida* (*Pdp*). The former was previously identified as *Photobacterium damsela* (and, earlier, as *Vibrio damsela*), and the latter as *Pasteurella piscicida*. Their close genetic relationship was demonstrated in 1995 through 16S rRNA sequencing and DNA-DNA hybridization analyses (Gauthier et al., 1995). However, the two subspecies are distinguishable by several phenotypical traits, with *Pdd* exhibiting flagellar motility, hemolytic activity, and the ability to grow at 37°C (Rivas et al., 2013b; Labella et al., 2018).

*Pdp* is the causative agent of photobacteriosis (pasteurellosis), a highly aggressive disease in aquaculture (Peèur Kazazić et al., 2019). *Pdd* is a halophilic bacterium that is widely distributed across global marine ecosystems; it is found both as a free-swimming microorganism (Ghinsberg et al., 1995; Klein et al., 2014) and as a pathogen, retaining its virulence for prolonged periods in oceans, estuarine environments, and sediments (Labella et al., 2017; Osorio et al., 2018). In recent years, it has been increasingly detected in a broader range of animal hosts, humans, and geographical areas (Terceti et al., 2016; Labella et al., 2017; Gouife et al., 2022).

### Literature selection strategy

This narrative review is based on a targeted literature search conducted in English across the PubMed, Scopus and Google Scholar databases. The literature search was initially performed in February 2025 and last updated in July 2026. The following search terms were used in combination: “*Photobacterium damselae*,” “*Photobacterium damselae* subsp. *damselae*,” “*Vibrio damsel*,” “virulence,” “outbreak,” “human,” “antimicrobial resistance,” “wildlife,” “aquaculture,” “heatwave,” and “climate change.” Because the available literature on *Photobacterium damselae* subsp. *damselae* (*Pdd*) remains relatively limited, no restrictions on publication date were applied to maximize the inclusion of relevant evidence. Titles and abstracts were initially screened for relevance, and the full texts of potentially eligible studies were subsequently assessed. Studies were included if they reported primary data on *Pdd* pathogenesis, host association, diagnostics, antimicrobial resistance, or ecology; studies referring exclusively to *Photobacterium damselae* subsp. *piscicida* without comparative relevance to *Pdd*, non-English language articles, preprints, and conference abstracts without a peer-reviewed full text were excluded. Retrieved articles were manually assessed for their relevance to the scope of this review, and the reference lists of selected papers were screened to identify additional relevant publications.

### Virulence factors

**FIGURE 1:**
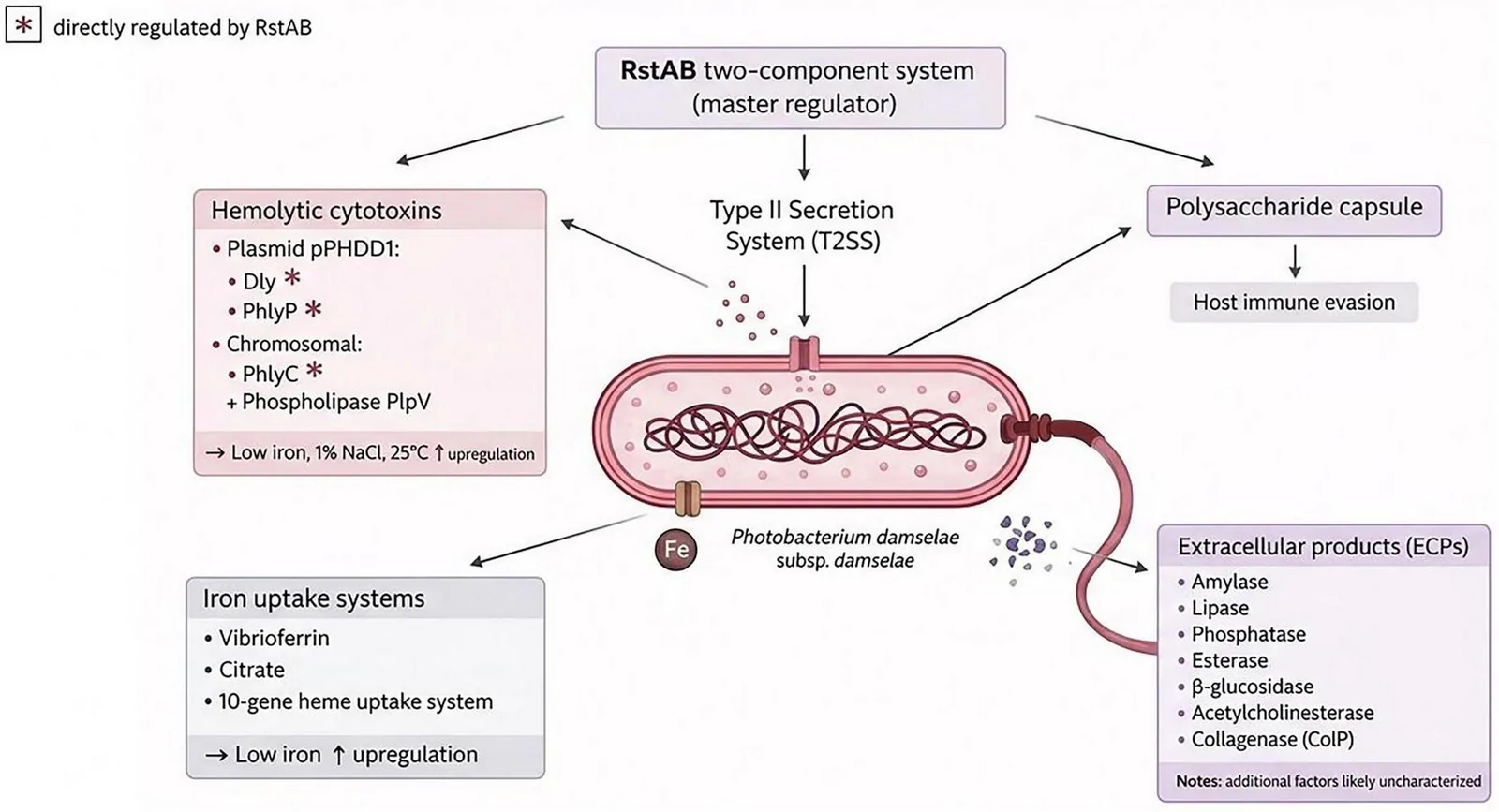
Schematic overview of *Pdd* virulence factors.

*Photobacterium damselae* subsp. *damselae* (*Pdd*) employs a variety of virulence factors to infect and evade host defense (Figure 1). Iron uptake systems are critical in iron-limited environments such as the host. This includes the siderophore vibrioferrin (compensated by citrate production when absent or inhibited) (Lemos and Balado, 2020) and a 10-gene heme uptake system (Río et al., 2005). Growth at 37°C leads to the downregulation of siderophore-specific receptors and ATP-binding cassette (ABC) transporters involved in iron uptake (Matanza and Osorio, 2020), whereas low iron availability upregulates hydroxamate-type siderophore systems (Fouz et al., 1997). The individual contributions of these systems to the pathogenesis require further elucidation (Osorio et al., 2018).

The best-characterized virulence factors are hemolytic cytotoxins. Plasmid pPHDD1 encodes thermolabile damselysin (Dly) and pore-forming phobalysin P (PhlyP) (Rivas et al., 2013b,^2015^), resulting in a higher hemolytic activity than that associated with the chromosomal *hlyAch* gene encoding phobalysin C (PhlyC) (Vences et al., 2017). These three hemolysins are secreted by the Type II Secretion System (T2SS) (Terceti et al., 2019). *Pdd* also produces the phospholipase PlpV, which is homologous to enzymes in other *Photobacterium* and *Vibrio*, and contributes to hemolysis and cytotoxicity (Vences et al., 2017). Hemolysin expression is environmentally regulated: low iron (Rivas et al., 2013a) and 1% NaCl (Barca et al., 2023) induce Dly, PhlyP, and PhlyC transcription. Several T2SS constituents are upregulated at 1% NaCl and 25 °C, a temperature associated with aquaculture outbreaks (Matanza and Osorio, 2018, 2020). Genetically, the two-component RstAB system is a master regulator of virulence and positively controls the expression of iron acquisition systems, cytotoxins, T2SS, and other proteins involved in bacterial pathogenesis (Terceti et al., 2019; Matanza et al., 2021). Transcriptomic profiling of RstAB has also revealed that a polysaccharide capsule is involved in evading immune responses and is necessary for full virulence (Matanza et al., 2021).

Extracellular products (ECPs) include enzymes such as amylase, lipase, phosphatases, β-glucosidase, esterase, and acetylcholinesterase (ichthyotoxin) but exhibit limited proteolytic activity (Fouz et al., 1993; Labella et al., 2010; Gouife et al., 2022). Collagenase ColP contributes modestly to pathogenicity (Vences et al., 2017). However, none of the detected enzyme activities alone explain the degree of observed toxicity, suggesting additional uncharacterized virulence molecules (Labella et al., 2010; Rivas et al., 2013b).

### Pathogenesis and transmission

Although the routes of host entry of *Photobacterium damselae* subsp. *damselae* (*Pdd*) remain poorly understood, available evidence indicates that it primarily infects aquatic animals through water (Fouz et al., 2000). Its transmission is influenced by environmental factors, including water temperature and salinity, and may be facilitated by stressful conditions associated with intensive farming, as well as by direct contact between animals (Fouz et al., 2000; Pedersen et al., 2009; Eissa et al., 2018). Additionally, *Pdd* exhibits a notable capacity to adhere to fish skin mucus, with adhesion capability differing across strains (Fouz et al., 2000; Khouadja et al., 2014). These findings suggest that skin likely serves as the primary portal of entry for the bacterium, especially if lesioned (Fouz et al., 2000; Gouife et al., 2022). Other transmission routes have recently been proposed in captivity, including contamination via food or human handling (Catanese and Grau, 2023), although these hypotheses require further investigation. Furthermore, *Pdd* may be a part of the normal microbiota of certain marine animals and could act as an opportunistic pathogen under stressful conditions or when host immune defenses are compromised (Catanese and Grau, 2023; Battistini et al., 2024; Bignami et al., 2026).

**FIGURE 2:**
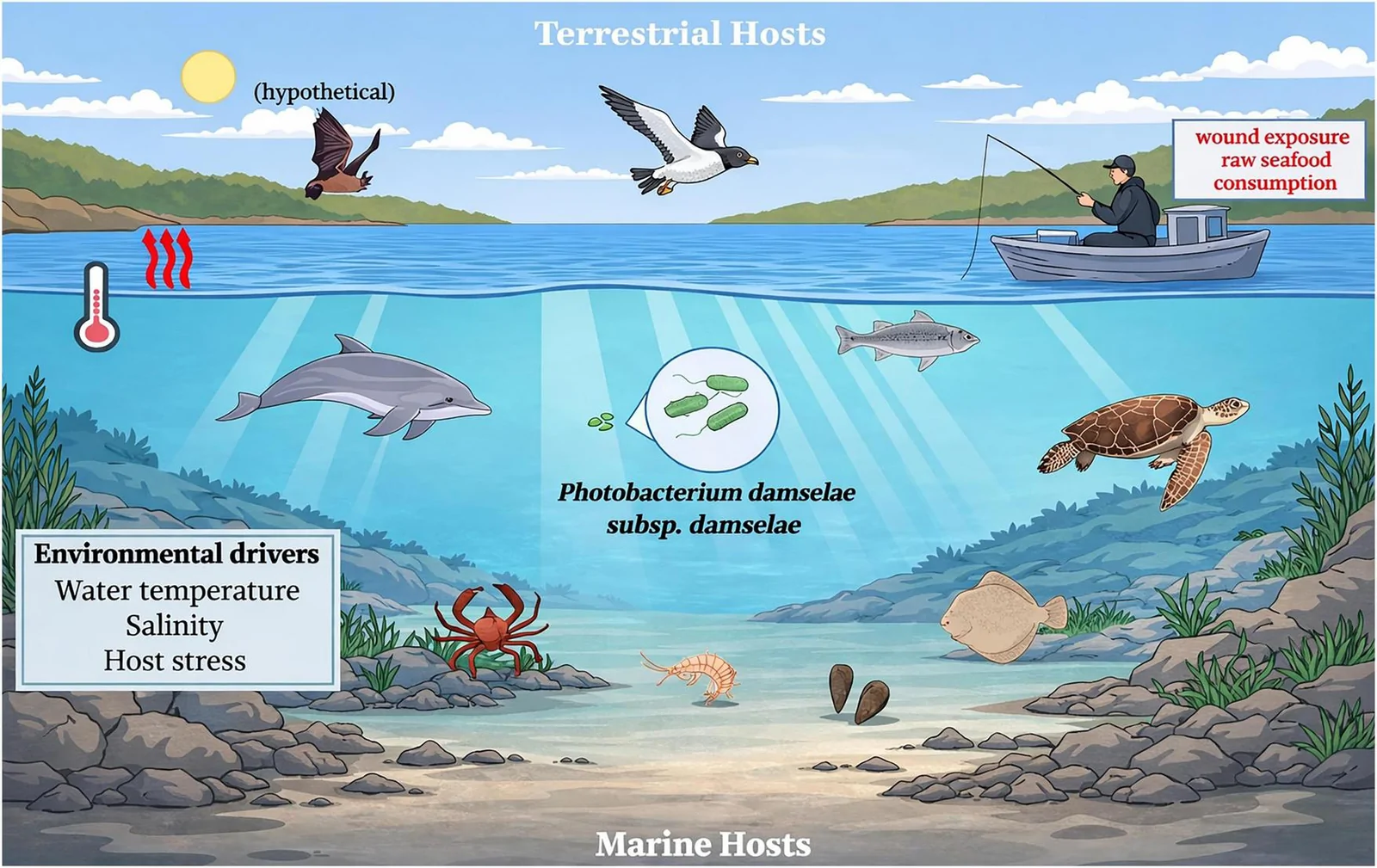
Conceptual representation of the ecological framework of *Pdd* across aquatic and terrestrial systems. *Pdd* has been isolated from seawater and a wide range of aquatic hosts, including fish, crustaceans, mollusks, sea turtles, and marine mammals. Reports of *Pdd* isolation from a seabird and its detection in piscivorous bats suggest that exposure may extend beyond strictly marine hosts, although their epidemiological role remains unclear. Human infections have been associated primarily with exposure of skin wounds to seawater and, less frequently, with the handling or consumption of raw or undercooked seafood. The figure illustrates potential ecological links and recognized routes of exposure based on the currently available evidence. Environmental drivers such as increased water temperature, salinity fluctuations, and host stress may influence bacterial persistence, proliferation, and host susceptibility.

*Pdd* has been reported in a broad range of hosts across multiple taxonomic groups, including both marine and terrestrial animals (Figure 2).

### Marine hosts

#### Fish, crustaceans, mollusks

Numerous commercially important fish species are recognized as primary hosts of *Pdd*, with outbreaks causing severe losses in aquaculture worldwide (Eissa et al., 2021; Gouife et al., 2022). In Europe, infections have been reported in farmed rainbow trout (*Oncorhynchus mykiss*) (Pedersen et al., 2009), seabass (*Dicentrarchus labrax*) (Bellos et al., 2015) and gilthead sea bream (*Sparus aurata*) (Pujalte et al., 2003), the latter two also affect African aquaculture (Khouadja et al., 2014). In Asia, outbreaks have involved black rockfish (*Sebastes schlegeli*) (Zhang et al., 2019) and half-smooth tongue sole (*Cynoglossus semilaevis*) (Shao et al., 2019). However, captive conditions are not a requisite for the infection, since *Pdd* has also been detected in wild fish populations (Khalil and Mesalhy, 2008; Trevisani et al., 2017; Petchimuthu et al., 2022). Additionally, strains of this subspecies have also been isolated from asymptomatic animals (Serracca et al., 2011; Rivas et al., 2013b; Trevisani et al., 2017).

Typical external lesions include extensive hemorrhagic areas and ulcers on the body surface, bleeding, abdominal distension with ascitic fluid and hepatomegaly (Fouz et al., 2000; Rivas et al., 2013b; Gouife et al., 2022; Bignami et al., 2026). Histopathological findings include severe congestion of blood vessels and focal infiltration of leukocytes in hepatic, splenic, and renal tissues; degeneration and necrosis of hepatocytes; moderate hyperplasia of melanomacrophage centers in the spleen and kidney; vacuolar degeneration or necrosis of lymphoid follicles; atrophy of hematopoietic tissue; and renal tubular degeneration (Gouife et al., 2022; Bignami et al., 2026).

Mollusks and crustaceans are also susceptible to infection (Lozano-León et al., 2003; Vaseeharan et al., 2007; Liu et al., 2016; Singaravel et al., 2020; Wang et al., 2020; Xie et al., 2021; Alolod et al., 2024; Chuchird et al., 2024). Crustaceans may show signs such as discoloration of the body surface and internal organs, loss of muscle elasticity, anorexia, and lethargy (Singaravel et al., 2020; Xie et al., 2021; Alolod et al., 2024: Chuchird et al., 2024). Pathogenicity data for mollusks remain limited.

#### Cetaceans and other marine mammals

*Pdd* infections have been primarily documented in cetaceans, including bottlenose dolphin (*Tursiops truncatus*), short-beaked common dolphin (*Delphinus delphis*), striped dolphin (*Stenella coeruleoalba*), Bryde’s whale (*Balaenoptera edeni*), and sperm whale (*Physeter macrocephalus*), among others (Rivas et al., 2013b; Alba et al., 2016; Morick et al., 2023; Battistini et al., 2024). Evidence from other marine mammal taxa remains limited; however, a presumptive systemic infection caused by *Pdd* was recently described for the first time in a spotted seal (*Phoca largha*) (Cha et al., 2025). This bacterium has also been isolated from asymptomatic cetaceans (Buck et al., 2006; Lee et al., 2018), suggesting an opportunistic pathogenic role. Supporting this hypothesis, a study on cetaceans stranded along the Ligurian coast (Italy) detected *Pdd* in 41.5% of samples, frequently associated with other bacterial and viral pathogens (Battistini et al., 2024).

In marine mammals, *Pdd* has been isolated from various tissues, including renal, pulmonary, hepatic, cerebral, and splenic (Battistini et al., 2024; Casalone et al., 2014; Gouife et al., 2022; Cha et al., 2025). The reported pathological findings include chronic pyogranulomatous pneumonia and splenic lymphoid depletion in bottlenose dolphins (Morick et al., 2023) as well as respiratory and intestinal congestion in spotted seals (Cha et al., 2025). Histologically, chronic inflammation, fibrinoid necrosis resulting from septic shock, edematous condition of the lungs, follicular depletion, and hyalinosis of the lymphoid tissue have been reported (Casalone et al., 2014; Morick et al., 2023; Battistini et al., 2024; Cha et al., 2025).

#### Sea turtles

*Pdd* has also been isolated from sea turtles, specifically *Dermochelys coriacea* (Obendorf et al., 1987; Oliver-Guimerà et al., 2019), *Chelonia mydas* (Aguirre et al., 1994), and *Caretta caretta* (Alba et al., 2016; Fichi et al., 2016). In *D. coriacea* postmortem examinations revealed fibrino-necrotizing enteritis and sepsis (Oliver-Guimerà et al., 2019), as well as valvular endocarditis where the bacterium was isolated from the endocardial thrombus (Obendorf et al., 1987). It should be noted that the earliest reports (Obendorf et al., 1987; Aguirre et al., 1994) predate the availability of reliable molecular methods used to distinguish *Pdd* from *Photobacterium damselae* subsp. *piscicida* (*Pdp*). Consequently, identification relied primarily on phenotypic characterization, and the reported subspecies assignments should therefore be interpreted with caution.

### Terrestrial hosts

#### Birds

A virulent strain of *Pdd* was recently isolated from a razorbill (*Alca torda*) stranded along the Italian coast, representing the first confirmed case of *Pdd* infection in a bird species (Minichino et al., 2026). The bird was found during a mass irruption and wrecking event in the Mediterranean Sea, likely associated with Atlantic storms and altered prey availability linked to environmental changes (Balestrieri et al., 2023). Individuals affected by this event showed severe debilitation, with starvation, weakness, and parasitism identified as the main causes of mortality (Loring Salmerón, 2023), potentially increasing their susceptibility to opportunistic infections. Although *Photobacterium damselae* has been previously detected in several avian species, including the brown pelican (*Pelecanus occidentalis*), common loon (*Gavia immer*), and great tit (*Parus major*), subspecies-level identification has not been performed (Ayala and Ogbunugafor, 2023). Given that *Pdp* growth is inhibited at 37 °C (Rivas et al., 2013b) and birds generally maintain higher body temperatures (Prinzinger et al., 1991), it could be hypothesized that at least some of the previous detections of *Photobacterium damselae* in avian species may have involved *Pdd* rather than *Pdp*, although this remains to be confirmed by subspecies-level characterization. These findings highlight the need for further investigation to clarify whether birds, and seabirds in particular, play any role in *Pdd* ecology and transmission.

#### Bats

Proteobacteria is a dominant phylum in the gastrointestinal microbiome of bats, which differs markedly from that of other terrestrial mammals (Huang et al., 2022). Although high abundances of Proteobacteria are often associated with dysbiosis in humans and mice, their functional role in bat health remains unclear (Jones et al., 2022). Recent studies have identified *Photobacterium* as a key genus in the gut microbiota of facultative piscivorous bats, particularly *Noctilio leporinus* and *Myotis vivesi*. The latter forage in marine lagoons and consume fish known to harbor symbiotic *Photobacterium* spp. (Aizpurua et al., 2021). The gut microbiome of *M. vivesi* is relatively specialized, with co-occurrence network analyses indicating that *Photobacterium damselae* is a major contributor to the community (Corduneanu et al., 2023). Although detection of *Photobacterium damselae* sequences in the gut microbiome of piscivorous bats does not confirm viable *Pdd* colonization or reservoir status, their dietary and ecological overlap with known *Pdd* hosts suggests that these species represent a potential host group warranting further investigation.

#### Humans

*Pdd* is recognized as an opportunistic human pathogen capable of causing severe and potentially fatal infections (Hundenborn et al., 2013; Matanza and Osorio, 2020; Caddia et al., 2022; Martin et al., 2022). To date, a total of 36 human *Pdd* cases have been reported globally, based on previous reviews (Hundenborn et al., 2013; Schröttner et al., 2020; Caddia et al., 2022) and two additional cases described subsequently (Martin et al., 2022; Schwartz et al., 2023). Nevertheless, several reports did not provide detailed information on the methods used for species identification, and some cases may have been influenced by previous taxonomic classifications or by the limited discriminatory power of older diagnostic approaches (Hundenborn et al., 2013; Schröttner et al., 2020).

The most common routes of infection involve exposure of skin wounds to saline or brackish waters (Hundenborn et al., 2013; Schröttner et al., 2020; Caddia et al., 2022), although cases following the consumption of raw fish or seafood have also been reported (Rivas et al., 2013b; Caddia et al., 2022). Other less common portals of entry include the urinary tract, as described in a case involving a pregnant woman (Alvarez et al., 2006). Reported infections have occurred predominantly in coastal regions of the United States, with severe wound infections mainly affecting males (Hundenborn et al., 2013; Schröttner et al., 2020; Caddia et al., 2022; Martin et al., 2022; Schwartz et al., 2023). The recurrent association with fish handling and fishing-related injuries suggests that *Pdd* may represent a potential occupational risk for individuals frequently exposed to marine environments (Hundenborn et al., 2013; Rivas et al., 2013b; Martin et al., 2022).

In several cases where comprehensive diagnostic approaches (bacteriological cultures, molecular identification, mass spectrometry, and sequencing methods) were applied, *Pdd* was identified as the primary etiological agent, although concurrent detection of *Vibrio* spp. was also reported, suggesting possible polymicrobial infections (Schröttner et al., 2020; Caddia et al., 2022). This possibility should be considered when interpreting clinical cases.

Despite the limited number of documented infections, *Pdd* may cause severe clinical outcomes. The most frequently reported manifestation is necrotizing fasciitis, which requires urgent surgical intervention and potentially progresses rapidly to multiorgan failure (Guimaraes et al., 2021). Predisposing factors such as advanced age and pre-existing conditions (diabetes mellitus, renal failure, cardiovascular disease) have been reported (Hundenborn et al., 2013; Guimaraes et al., 2021; Martin et al., 2022); however, *Pdd* has also been isolated from healthy individuals (Alvarez et al., 2006; Chochlakis et al., 2019). Human body temperature is a stressful condition for *Pdd*, affecting its viability (Matanza and Osorio, 2020). Although growth at 37°C downregulates virulence factors, chemotaxis, and flagellar motility, the three major cytotoxin genes (*dly*, *hlyAch, hlyApl*) remain highly expressed; the resulting accumulation of their products in host fluids may exacerbate tissue damage even in the absence of bacterial replication (Matanza and Osorio, 2020).

Overall, although confirmed human cases are relatively rare, the severity of reported cases highlights the importance of preventive measures and clinical awareness following wound exposure to marine environments and consumption of raw fish or seafood. Furthermore, the number of human infections caused by *Pdd* may be underestimated due to underreporting and the missed diagnosis of *Pdd* because testing is preferentially directed toward other, better-known marine bacterial pathogens, such as *Vibrio* spp.

### Diagnostic methods

*Photobacterium damselae* infection is diagnosed using traditional microbiological methods, such as bacteriological cultures on TSA, blood agar (both supplemented with 1–2% NaCl), TCBS, or 2216 marine agar, with incubation at room temperature for 24–96 h (Andreoni and Magnani, 2014; Gouife et al., 2022). Differentiation between *Photobacterium damselae* subsp. *damselae* (*Pdd*) and *Photobacterium damselae* subsp. *piscicida* (*Pdp*) requires additional biochemical, molecular, or serological assays. *Pdd* is phenotypically distinguished from *Pdp* by positive motility and urease activity, as well as by its ability to grow on blood agar, TCBS agar, and at 37 °C. Commercial identification systems, such as API 20E (bioMérieux), together with serological methods, including agglutination tests and ELISA, can further assist subspecies identification (Andreoni and Magnani, 2014). However, atypical strains may lead to misidentification (Rivas et al., 2013b; Andreoni and Magnani, 2014), particularly in clinical settings where *Photobacterium* spp. is not routinely considered. Consequently, phenotypic characterization should be regarded as presumptive, and subspecies identification should ideally be confirmed using molecular methods, including hybridization, DNA microarray, PCR, and molecular typing. PCR assays commonly target the 16S rRNA gene, and increased specificity is obtained using markers such as *toxR* (Martins et al., 2015). For subspecies differentiation, multiplex PCR combining 16S rRNA primers (CAR-1, CAR-2) with the *ureC* gene, which is present in *Pdd* but absent in *Pdp*, is widely recommended (Osorio et al., 2000; Minichino et al., 2026). Additionally, a single-step PCR targeting SNPs within the *bamB* gene enables detection, quantification, and subspecies identification (Carraro et al., 2018). Genotyping approaches, such as the MLST scheme proposed by Alba et al. (2016), based on six genes (*gyrB, toxR, glp, metG, pnt*, and *pyrC*), have also proven effective in resolving phylogenetic relationships among *Pdd* strains from diverse hosts and geographic regions.

Finally, rapid identification can also be achieved using MALDI-TOF MS coupled with ClinProTools software, although manual adjustment of the spectral peaks may be required (Pérez-Sancho et al., 2016).

The choice of diagnostic approach should ultimately depend on the purpose of the investigation and the level of resolution required. For routine diagnosis in clinical, veterinary, and aquaculture settings, initial isolation by conventional bacteriological culture or, where available, rapid identification by MALDI-TOF MS should be followed by molecular confirmation to ensure accurate subspecies identification. For wildlife surveillance and environmental monitoring, molecular methods offer the additional advantage of greater sensitivity, particularly when bacterial loads are low or when bacterial isolation is compromised by challenging field conditions and poor sample quality (Zhen et al., 2026). Among these, the multiplex PCR assay combining the 16S rRNA gene with the *ureC* gene represents a practical, accurate, and cost-effective approach for subspecies identification across all contexts, as reported by Osorio et al. (2000), and by our previous study (Minichino et al., 2026). Following subspecies identification, additional PCR assays targeting the major virulence genes (*dly*, *hlyApl*, *hlyAch*) can be performed to further characterize the pathogenic potential of the isolate. This information may be particularly valuable in clinical and veterinary settings for assessing the virulence profile of the infecting strain. For epidemiological investigations and research purposes, higher-resolution genotyping methods, such as MLST, and whole-genome sequencing, are recommended to investigate strain diversity, phylogenetic relationships, and transmission pathways.

Although molecular and proteomic tools have substantially improved diagnostic accuracy, their adoption remains uneven across laboratories and host contexts. Consequently, despite the broad host range of *Pdd*, molecular diagnostics and virulence profiling have been applied in relatively few studies (Table 1), limiting our understanding of transmission pathways, host associations, and the true epidemiological relevance of this pathogen.

**TABLE 1:** Host species (excluding fish) in which *Pdd* was identified using PCR-based molecular diagnostics, along with detected virulence factors.

Host species	Common name	Host taxon	Location	PCR target	Virulence factors (genes)	Detection context	References
*Mytilus galloprovincialis*	Mediterranean mussel	Mollusca	Spain	16S rRNA + *ureC*	nr	Asymptomatic detection	Lozano-León et al. (2003)
*Scylla paramamosain*	Mud crab	Crustacea	China	16S rRNA + *ureC*	nr	Confirmed disease causation	Xie et al. (2021)
*Litopenaeus vannamei*	Pacific white shrimp	Crustacea	China	16S rRNA + *toxR*	nr	Confirmed disease causation	Wang et al. (2020)
*Litopenaeus vannamei*	Pacific white shrimp	Crustacea	Thailand	16S rRNA + *ureC*	nr	Confirmed disease causation	Chuchird et al. (2024)
*Penaeus monodon*	Black tiger shrimp	Crustacea	India	16S rRNA + *ureC*	nr	Confirmed disease causation	Vaseeharan et al. (2007)
*Penaeus japonicus*	Kuruma shrimp	Crustacea	Japan	16S rRNA + *ureC*	*hlyAch, PlpV*	Confirmed disease causation	Alolod et al. (2024)
*Exopalaemon carinicauda*	Ridgetail prawn	Crustacea	nr	16S rRNA + *toxR*	nr	Confirmed disease causation	Liu et al. (2016)
*Tursiops truncatus*	Bottlenose dolphin	Cetacea	Israel	16S rRNA + MLST*	*hlyApl, dly*	Confirmed disease causation	Morick et al. (2023)
*Tursiops truncatus*	Bottlenose dolphin	Cetacea	Italy	16S rRNA + *ureC*	*hlyAch, hlyApl, dly*	Isolation from diseased animals	Battistini et al. (2024)
*Stenella coeruleoalba*	Striped dolphin	Cetacea			*hlyAch, hlyApl, dly*		
*Stenella coeruleoalba*	Striped dolphin	Cetacea	Italy	MLST*	*hlyAch, hlyApl, dly*		
*Delphinus delphis*	Short-beaked common dolphin	Cetacea			negative	Isolation from diseased animals	Alba et al. (2016)
*Tursiops truncatus*	Bottlenose dolphin	Cetacea			*hlyAch*		
*Physeter macrocephalus*	Sperm whale	Cetacea			*hlyAch*		
*Caretta caretta*	Loggerhead sea turtle	Reptilia	Italy		*hlyAch*		
*Alca torda*	Razorbill	Aves	Italy	16S rRNA + *ureC*	*hlyAch, hlyApl, dly*	Confirmed disease causation	Minichino et al. (2026)

Context of detection was classified into three categories: (i) confirmed disease causation, supported by isolation and consistent clinical/pathological findings; (ii) isolation from diseased animals, where *Pdd* was isolated from a diseased animal without an established causal role (e.g., polymicrobial infection or concurrent isolation with other pathogens); and (iii) asymptomatic detection, where *Pdd* was isolated from apparently clinically healthy individuals. *nr* = not reported. *MLST = multilocus sequence typing following the scheme by Alba et al. (2016).

### Antimicrobial resistance pattern and alternative experimental strategies to antibiotics against *Photobacterium damselae* subsp. *damselae*

The prolonged use of antibiotics in aquaculture has led to the development of resistance in various strains of *Photobacterium damselae* subsp. *damselae* (*Pdd*), underscoring the importance of their prudent and responsible use (Vences et al., 2020; Xie et al., 2021). Indeed, numerous cases of multiple antibiotic resistance have been documented for this subspecies. The antimicrobial resistance (AMR) profiles reported for *Pdd* have been mainly investigated in isolates recovered from fish and seafood sources. Specifically, resistance has been observed against commonly used antibiotics acting on diverse bacterial targets, including β-lactams (e.g., penicillins such as ampicillin and amoxicillin), tetracyclines, macrolides (e.g., erythromycin), aminoglycosides (e.g., streptomycin, gentamicin), phenicols (e.g., chloramphenicol), sulfonamides, and (fluoro)quinolones (e.g., oxolinic acid and enrofloxacin), among others (Fouz et al., 1992; Han et al., 2009; Zhang et al., 2011; Chiu et al., 2013; Shao et al., 2019; Petchimuthu et al., 2022; Weawsawang et al., 2024).

Resistance genes can be transferred via pAQU-type plasmids, which represent the best-characterized resistance determinants in *Pdd*. The prototype plasmid pAQU1, isolated from a tetracycline-resistant *Pdd* strain collected in Japan, carries seven different resistance genes and is transferable to *Escherichia coli* and *Photobacterium damselae* subsp. *piscicida* through conjugation, suggesting potential horizontal gene transfer among bacterial species (Nonaka et al., 2012; Vences et al., 2020). Among these resistance genes, the tandem *mef*(C)-*mph*(G) gene pair confers high-level macrolide resistance (Nonaka et al., 2015). pAQU-type plasmids are not confined to East Asia: structurally related variants, carrying different combinations of resistance genes, have also been reported in Mediterranean aquaculture and, more recently, in South Korea, indicating repeated independent acquisition by distinct *Pdd* lineages rather than the spread of a single resistant clone (Vences et al., 2020; Kim et al., 2026). Plasmids characteristic of isolates from the Black Sea also showed the acquisition of new genes, such as *strAB*, and high transfer frequency, raising concerns about their potential contribution to the resistome in aquatic and terrestrial environments (Vences et al., 2020). Moreover, pAQU1 can persist even under low levels of antibiotic selective pressure (Bien et al., 2015). However, the majority of currently available detailed AMR data derive from aquaculture-associated isolates, and comparable information for wildlife, environmental, and human-derived strains remains lacking. Together, these findings underscore the need to explore alternative strategies for preventing and controlling *Pdd* infections, including vaccines, antimicrobial peptides, bacteriophages, probiotics, prebiotics, and immunostimulants.

Several alternative strategies have been investigated to reduce the impact of *Pdd* infections, particularly in aquaculture. However, most of these approaches remain at the experimental stage, and their practical application is still limited by the lack of field validation.

Among these, vaccination has been evaluated as a preventive strategy against *Pdd*, although no commercial vaccine is currently available. In 2022, a novel oral biofilm vaccine combined with chitosan particles showed a 60% survival rate in giant groupers (*Epinephelus lanceolatus*) challenged with *Pdd*, along with increased phagocytosis, IgM levels, and upregulation of immune genes (Su and Chen, 2022). Subsequently, a formalin-inactivated vaccine combined with Montanide ISA 70 VG showed a survival rate of 40–55% in *Lates calcarifer* (Weawsawang et al., 2024); the lower rate compared to the previous study may reflect differences in adjuvant, host species, or challenge protocol.

Other biological approaches have also yielded encouraging results. Probiotics such as *Shewanella* species (*S. putrefaciens* and *S. baltica*) have improved fish survival and growth in the presence of *Pdd* (Díaz-Rosales et al., 2009; García de la Banda et al., 2010). Bacteriophages offer a viable alternative to antibiotics owing to their selective lytic action against specific bacteria (Tiwari et al., 2014). *Myoviridae* bacteriophage *Phda1* inhibits the growth and histamine production of *Pdd* (Yamaki et al., 2015). Additionally, the *Phage-I* bacteriophage, also from the *Myoviridae* family, exhibits lytic activity against *Pdd*, achieving an 83.33% survival rate in silver pomfret (*Pampus argenteus*) after challenge (Li et al., 2024). Moreover, the combination of bacteriophages and antibiotics (phage–antibiotic synergy) proved to be highly effective against *Pdd*. For example, the use of *Pdd* phage *TEMp-D1* and four antibiotics (oxytetracycline, florfenicol, sulfadiazine/trimethoprim, and enrofloxacin) resulted in significantly high inhibition rates (Eroğlu and Yaşa, 2025).

Natural products have mainly been investigated as dietary immunostimulants rather than direct antimicrobial agents. For instance, ginger (*Zingiber officinale*) extract, alone or in combination with thyme (*Thymus vulgaris*) and garlic (*Allium sativum*), has been shown to enhance growth, antioxidant activity, and resistance to *Photobacterium damselae* infection in *Penaeus vannamei* and *Sparidentex hasta* (Jahanjoo et al., 2018; Shahraki et al., 2021). Algal extracts, such as brown seaweed (*Fucus virsoides*) and *Ulva ohnoi*, also exhibit inhibitory effects on *Pdd* and modulate immune responses in fish (Rizzo et al., 2017; Fumanal et al., 2020). A recent study (Lee et al., 2022) on cobia (*Rachycentron canadum*) demonstrated that water extracted from the alga *Sarcodia suae* (SSWE) increased the expression of cytokines (IL-1β, IL-6, IL-10, IL-12, and TNF-α) and chemokines (IL-8) *in vitro* and *in vivo*. Intraperitoneal injection of SSWE followed by a challenge with *Pdd* delayed mortality in infected animals and elevated Th1-type cytokines, suggesting SSWE’s potential as an immunostimulant and vaccine adjuvant for fish (Lee et al., 2022).

Among emerging alternatives, antimicrobial peptides (AMPs) have also attracted increasing interest, although limited experimental evidence is currently available for *Pdd*. Histone-derived peptides from silver pomfret (*Pampus argenteus*), namely spH4 and spH2A.2, have shown *in vitro* antibacterial activity against *Pdd* by disrupting bacterial cell integrity (Huang et al., 2024). Notably, whereas spH4 showed no detectable cytotoxicity toward host cells, spH2A.2 displayed cytotoxic effects, underscoring the need to balance antimicrobial efficacy with host safety when developing peptide-based therapeutics. Likewise, studies on the closely related *Photobacterium damselae* subsp. *piscicida* have shown that exposure to AMPs triggers adaptive stress-response pathways, providing mechanistic insights that may inform the rational development of peptide-based antimicrobial strategies (Tsai et al., 2015).

Overall, while vaccines, probiotics, bacteriophages, natural immunostimulants, and AMPs represent promising alternatives or complements to antibiotic therapy, current evidence is largely derived from laboratory or small-scale experimental studies. Further research is needed to validate their safety, efficacy, delivery strategies, and economic feasibility before their widespread application in aquaculture.

### Genomic resources and bioinformatics perspectives

Understanding the molecular basis and dynamics that determine the adaptability and ability of *Photobacterium damselae* subsp. *damselae* (*Pdd*) to infect a wide range of hosts is essential to unravel its ecological complexity. The development of sequencing techniques has provided a decisive boost in the study of these mechanisms (Ekblom and Galindo, 2011). Nowadays, the ability to collect and classify several genomes, as well as to analyze a large amount of molecular data using public databases, is an essential tool for furthering our knowledge. Analyzing genomic variability, plasmid characteristics, and gene sequences in relation to virulence or antimicrobial resistance has opened highly significant prospects for the scientific community. Through these approaches, bioinformatics and genomic analysis allow the outlining of bacterial epidemiological networks (Suster et al., 2024), besides building a suitable knowledge base for the development of innovative diagnostic tests based on specific primers (Avershina et al., 2023). This knowledge is crucial for the implementation of predictive algorithms capable of describing and predicting the ecological evolution of microorganisms (Sironi and Kaderali, 2021). The identification and characterization of genes related to pathogenicity are key steps in categorizing and distinguishing strains based on their pathogenic potential, whereas the analysis of strains carrying AMR-associated genes is essential for monitoring the spread of resistance determinants and defining targeted control strategies based on genomic evidence (Wheeler et al., 2023).

Based on these conceptual advances, studies of real *Pdd* populations provide concrete examples of genomic variability and multiclonality. Ribotyping studies and pulsed-field gel electrophoresis (PFGE) revealed a high degree of heterogeneity among the different isolates: in one study, 26 isolates were classified into 17 distinct ribotypes, while in another, 16 isolates each showed a unique PFGE profile (Pedersen et al., 1997, 2009). Analogous observations have also been reported in other geographical areas, confirming that high genetic heterogeneity characterizes strains originating from the same region (Terceti et al., 2018). Rivas et al. (2014) showed high variability among different strains in the region of chromosome I close to the *hlyAch* gene, responsible for the PhlyC toxin, in which each strain showed strain-specific gene repertoires. The main difference between the two chromosomes, apart from their size, is their degree of conservation. Chromosome I is the most conserved and is believed to contain some of the “core” genes essential for replication, transcription, translation, and central metabolism (Thompson et al., 2009). Chromosome II shows high variability between strains of the same species and between different species, due to regions rich in genes acquired through horizontal transfer, mobile elements, and accessory functions. This variability determines the genetic plasticity of the *Photobacterium* genus, generating phenotypic differences between strains, including metabolic capabilities, environmental adaptations, and potential virulence characteristics (Vesth et al., 2010; Lukjancenko and Ussery, 2014). These results fit into a broader context of genomic diversity within the *Photobacterium* genus: a recent comparative genomics study highlighted a high degree of heterogeneity, with the main genome of the genus comprising 1,232 genes, after comparing 35 genomes (Machado and Gram, 2017). According to this evidence, Terceti et al. (2018) provided evidence supporting the presence of unique gene combinations in each *Pdd* strain analyzed. Differences in annotation criteria, assembly quality, and typing methods can complicate comparisons between studies. Genomic variability and the dynamic nature of the accessory genome hinder phylogenetic comparisons and the interpretation of genetic variability. These characteristics make *Pdd* strains adaptable to different conditions and underscore the need for surveillance programs with comprehensive sequencing, standardized analysis, and monitoring of variable clusters, which are essential for understanding population genetics, detecting new genotypes, and supporting control strategies based on genomic evidence.

To assess the current status of genomic research on *Pdd*, the NCBI Genomes database was searched on October 29, 2025, using the taxonomic term “*Photobacterium damselae* subsp. *damselae.*” Genomes were included if their taxonomic assignment was confirmed according to the NCBI Taxonomy database, complete BioSample metadata (host, geographic origin, and isolation date) were available, the year of isolation was 2010 or later, and the assembly quality reached at least the Contig level according to the NCBI Assembly Level classification. Genomes isolated before 2010, records lacking essential metadata, and duplicate assemblies of the same isolate were excluded. When multiple assemblies were available for a single isolate, the most recent RefSeq assembly was retained; if no RefSeq assembly was available, the corresponding GenBank assembly was selected. The final dataset comprised 27 genomes, classified according to the NCBI Assembly Level as Complete (*n* = 14, 51.9%), Scaffold (*n* = 5, 18.5%), and Contig (*n* = 8, 29.6%). Based on their source of isolation, 26 genomes (96.3%) originated from farmed fish, marine environments, or aquaculture settings, whereas one genome (3.7%) was recovered from a human clinical isolate. A comprehensive description of all genomes included in the analysis, together with their NCBI accession numbers and associated metadata, is provided in Supplementary Table 1.

**FIGURE 3:**
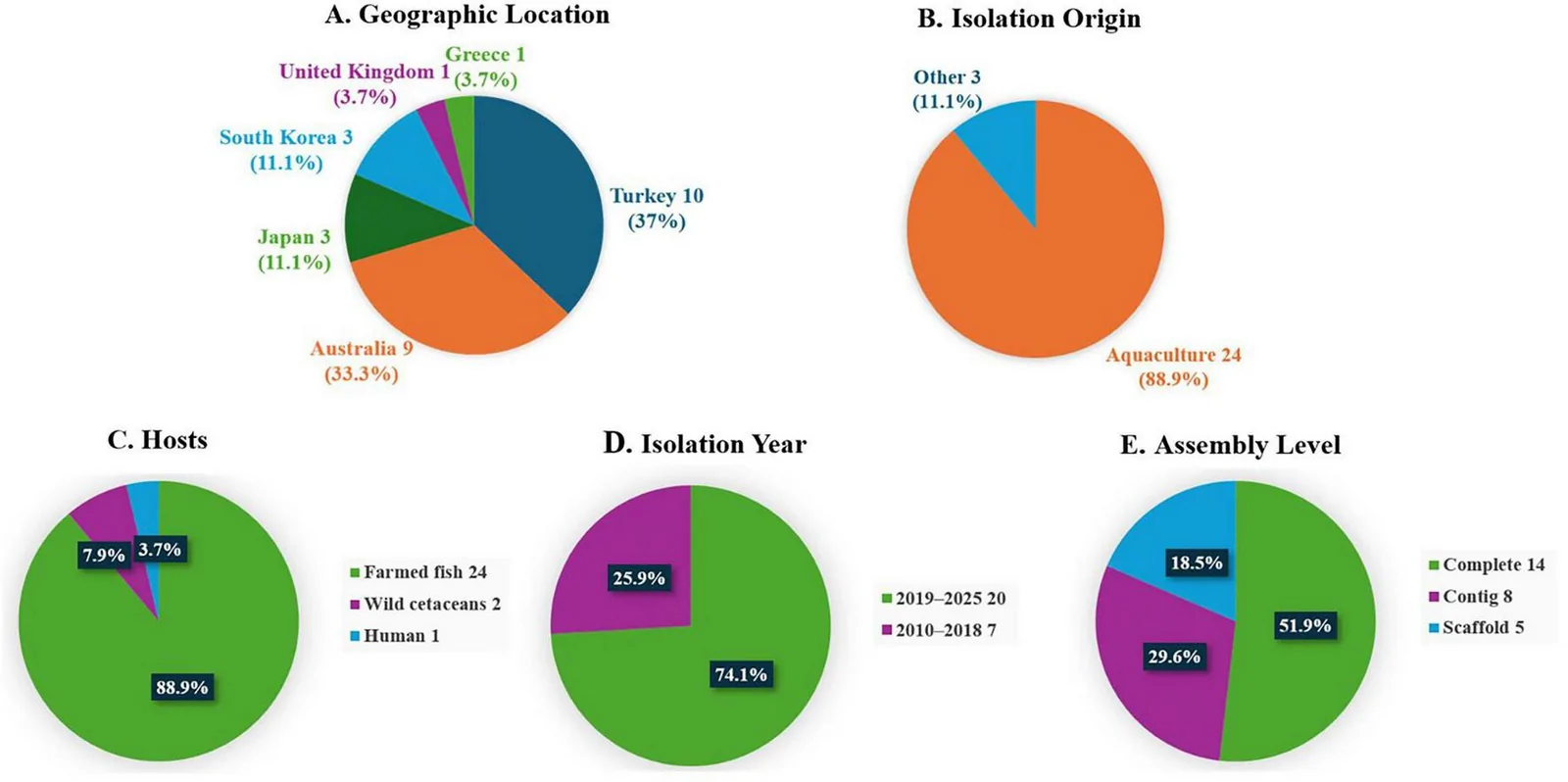
Overview of the currently available *Pdd* genome assemblies included in this review. Pie charts show the distribution of genomes according to **(A)** geographic location, **(B)** isolation origin, **(C)** host, **(D)** year of isolation, and **(E)** genome assembly level.

Among the 27 genomes included in the analysis, the distribution by source revealed a strong predominance of aquaculture-associated isolates. Twenty-four genomes (88.9%) were obtained from fish farming environments, whereas only three genomes (11.1%) originated from non-aquaculture sources, including two isolates from wild marine cetaceans and one from a human clinical case. A similar pattern was observed in the geographic distribution, highlighting a strong concentration of genomic studies in countries with intensive aquaculture production. The analyzed genomes originated from six countries: Turkey (*n* = 10, 37.0%), Australia (*n* = 9, 33.3%), Japan (*n* = 3, 11.1%), South Korea (*n* = 3, 11.1%), the United Kingdom (*n* = 1, 3.7%), and Greece (*n* = 1, 3.7%). Notably, Turkey, Australia, and Japan together accounted for 22 of the 27 genomes (81.5%), regions with well-established aquaculture industries (Aydın et al., 2025; Mechaly et al., 2025). Details on the number of genomes per host, geographic location, isolation origin assembly level, and year are provided in Figure 3.

As previously noted, only 14 genomes meeting our criteria in the database are assembled at the “complete” level. Different assembly strategies and curation levels can produce fragmented data, hindering robust phylogenetic analysis and efficient genomic study (Pedersen et al., 1997; Zheng and Sankoff, 2016). The comparability of the dataset is influenced by the level of fragmentation and the variability of the assembly and annotation pipelines (Terceti et al., 2018). In genomic studies, type strains are essential taxonomic and genomic references. For *Pdd*, the reference strain is designated as ATCC 33539, CAIM 331, CCUG 13626, CDC 2588-80, CIP 102761, DSM 7482, IFO 15633, LMG 7892, NBRC 15633, and NCTC 11647 (Göker et al., 2025). Its complete and annotated genome supports species definition and comparative analyses, representing the core genome and accessory elements, including plasmids pPHDD1 and pPHDD203. The genome of CIP 102761, approximately 5.05 Mbp in size and with a GC content of 40.7%, is considered complete and of such high quality that it is one of the most widely used reference strains compared to many recent isolates, which often have incomplete or fragmented sequences (Vences et al., 2017). However, CIP 102761 has limitations: accessory elements such as the pPHDD203 plasmid with a type III secretion system may influence the interpretation of virulence, and comparisons based solely on archetypal strains may not capture the diversity of accessory genes and antigenic clusters in emerging strains. Nevertheless, these strains remain essential for comparative analyses, surveillance, and identification of virulence determinants, and their integration with recent isolates allows for a more realistic epidemiological and functional picture.

In this context, it is necessary to expand the sequencing and annotation of new isolates, diversifying hosts, and geographical areas. The use of long-read technologies, shared criteria for genome validation, and standardized bioinformatics pipelines would increase the comparability of results and strengthen functional analyses (Kim et al., 2024). These efforts could enable the creation of comprehensive comparative genomic datasets, thereby providing an integrated understanding of the molecular mechanisms underlying the environmental adaptation and zoonotic potential of *Pdd*.

### The twofold dark side of climate change: direct and indirect drivers of increased *Photobacterium damselae* subsp. *damselae* infection risk

Climate change profoundly alters marine ecosystems, increasing the morbidity and mortality of wildlife populations through the emergence and re-emergence of infectious diseases (Aguirre and Tabor, 2008). Since the pre-industrial period, global ocean surface temperatures have increased substantially, with accelerated warming trends over recent decades (von Schuckmann et al., 2024). Future climate scenarios predict the simultaneous occurrence of elevated seawater temperatures, hypoxia, acidification, salinity fluctuations, and pollution, creating multi-stressor environments with synergistic negative effects on marine organisms (Gunderson et al., 2016; Spinos et al., 2024; Smith et al., 2023). These stressors impair immune function, alter behavior, and promote energetic imbalances, thereby increasing host susceptibility to infection and contributing to mass mortality events in marine wildlife and aquaculture systems (Smith et al., 2023; Stillman et al., 2025). Climate-driven environmental changes may also influence pathogen dynamics by modifying host distributions, facilitating novel host–pathogen encounters, and inducing dysbiosis in the gut microbiome, which reduces microbial diversity and weakens colonization resistance against opportunistic pathogens (Fuller et al., 2012; Xiong et al., 2019; Carlson et al., 2022; Suzzi et al., 2023; Liao et al., 2024; Okon et al., 2024). Beyond host-associated dysbiosis, climate change is increasingly recognized as a driver of a broader “silent microbial shift,” characterized by gradual and often imperceptible reorganization of microbial communities at the ecosystem level (Naga et al., 2025). Such changes may indirectly influence disease risk by modifying the background microbial landscape in which opportunistic pathogens operate. In this context, temperature-sensitive marine bacteria, including members of the *Vibrionaceae*, have been proposed as informative indicators of broader ecosystem responses to environmental change (Zhang et al., 2023; Naga et al., 2025).

Rising sea surface temperatures, especially those above 15°C, are considered a significant factor driving the abundance and spread of *Vibrionaceae* species (Bellos et al., 2015), ultimately increasing the susceptibility of marine organisms to bacterial pathogens. This may be the case for infections caused by both subspecies of *Photobacterium damselae*. *Photobacterium damselae* subsp. *piscicida* displays a seasonal prevalence, with outbreaks typically occurring when water temperatures exceed 23 °C, indicating that thermal stress facilitates the onset of photobacteriosis (Spinos et al., 2024). As for *Photobacterium damselae* subsp. *damselae* (*Pdd*), experimental studies indicate that temperature directly modulates the bacterial physiology. At approximately 25 °C, *Pdd* upregulates genes involved in nutrient acquisition, metabolism, chemotaxis, motility, and virulence, promoting bacterial proliferation and enhanced evasion of host immune responses (Matanza and Osorio, 2018). However, the extent to which these findings translate into consistent epidemiological patterns in natural settings remains uncertain.

Considering mortality outbreaks reported in aquaculture settings, which are more likely to be detected and investigated than cases in wildlife, an apparent association between *Pdd* mortality events and warm periods emerges (Table 2), although environmental data are often incomplete or unreported. This pattern has been reported across multiple regions and host species of farmed fish, with outbreaks frequently occurring during summer months or unusually warm seasons. A comparable trend has been noted in crustaceans, with mass mortalities reported during periods of elevated temperature (Wang et al., 2020; Xie et al., 2021; Alolod et al., 2024) or reduced salinity (Chuchird et al., 2024) in shrimp aquaculture (Table 2). Experimental evidence further suggests that in *Penaeus monodon*, abrupt temperature fluctuations can impair immune function, as reflected by reduced hemocyte activity, phagocytosis, and antioxidant enzyme defenses, thereby increasing susceptibility to *Pdd* infection (Wang and Chen, 2006). The Mediterranean Sea offers a further, more circumstantial line of evidence. As a recognized hotspot of climate change, with warming rates exceeding the global average and frequent marine heatwaves (von Schuckmann et al., 2024), it has also been the source of most *Pdd* infections reported in marine wildlife beyond fish, including dolphins, turtles, seabirds, and sporadic human cases (Lozano-León et al., 2003; Casalone et al., 2014; Caddia et al., 2022; Morick et al., 2023; Minichino et al., 2026). This concentration of reports, however, may partly reflect more intensive research, stranding-network, and diagnostic activity in the region rather than a genuinely higher underlying risk. Overall, the frequent lack of environmental data, particularly water temperature, together with potential differences in surveillance effort among regions limits the interpretation of *Pdd* outbreak patterns and their association with climatic factors.

**TABLE 2:** Reported *Pdd* mortality outbreaks in aquaculture settings.

Affected species	Location	Water temperature (°C)	Month or season	References
Gilt-head seabream (*Sparus aurata*)	Atlantic Ocean, Spain	26	Summer	Vera et al. (1991)
Turbot (*Scophthalmus maximus)*	Atlantic Ocean, Spain	22–24	Summer	Fouz et al. (1992)
Redbanded seabream *(Pagrus auriga)*	Mediterranean Sea, Spain	nr	April-June, October-December	Labella et al. (2011)
White seabream *(Diplodus sargus)*			August	
Meager *(Argyrosomus regius)*			August	
Rainbow trout *(Oncorhynchus mykiss)*	Atlantic Ocean, Denmark	nr	June-September	Pedersen et al. (2009)
European seabass *(Dicentrarchus labrax)*	Mediterranean Sea, Tunisia	20–25	Spring-Summer	Khouadja et al. (2014)
Gilt-head seabream *(Sparus aurata)*				
European seabass *(Dicentrarchus labrax)*	Mediterranean Sea, Egypt	29–31	Summer	Battistini et al. (2024)
Gilthead seabream *(Sparus aurata)*				
Thin-lipped mullet *(Liza ramada)*				
European seabass *(Dicentrarchus labrax)*	Mediterranean Sea, Greece	19–25	May-August	Bellos et al. (2015)
Half-smooth tongue sole *(Cynoglossus semilaevis)*	Pacific Ocean, China	nr	March	Zhang et al. (2011)
Pacific white shrimp *(Penaeus vannamei)*	Pacific Ocean, China	nr	June	Wang et al. (2020)
Mud crab *(Scylla paramamosain)*	Pacific Ocean, China	nr	May	Xie et al. (2021)
Kuruma shrimp *(Penaeus japonicus)*	Pacific Ocean, Japan	nr	August-September	Alolod et al. (2024)
Pacific white shrimp *(Penaeus vannamei)*	Pacific Ocean, Thailand	nr	Rainy season	Chuchird et al. (2024)

Only cases in which *Pdd* was identified as the primary etiological agent and for which environmental information (water temperature and/or month or season) was available were included. Data are reported as described in the original studies to avoid over-interpretation. nr, not reported.

## Discussion

*Photobacterium damselae* subsp. *damselae* (*Pdd*), with its broad host range and zoonotic potential, is of increasing concern within a One Health framework. However, its epidemiological role varies considerably across host species and ecological contexts. While *Pdd* has been identified as the primary etiological agent of disease in several aquatic species, in other cases it has been isolated from diseased animals without definitive evidence of causality, detected as part of polymicrobial infections, or isolated from apparently healthy hosts. Consequently, the literature reviewed here supports characterizing *Pdd* predominantly as an opportunistic pathogen whose pathogenicity depends on the interplay between bacterial virulence, host susceptibility, and environmental conditions rather than the mere presence of the pathogen.

An important consideration emerging from the available literature concerns the interpretation of the apparent increase in reports involving *Pdd*. Although infections have been documented in an expanding range of hosts and geographical areas, the current evidence precludes a definitive conclusion on whether this reflects a true epidemiological emergence. Instead, the observed increase may result from multiple factors, including improved diagnostic methods and a broader use of molecular identification, increased surveillance and reporting, or genuine ecological changes affecting pathogen distribution and host susceptibility. Moreover, clinical signs are often non-specific, increasing the risk of misdiagnosis and underreporting, with a consequential publication bias. In wildlife, most reports consist of isolated case descriptions or opportunistic or incidental findings rather than structured epidemiological investigations, making it difficult to determine the true prevalence of infection or the epidemiological significance of newly reported hosts. Similarly, the limited number of confirmed human cases may partly reflect underdiagnosis, especially because *Pdd* can be misidentified as other marine bacteria when molecular confirmation is not performed. Likewise, recent detections in a seabird and a marine mammal beyond cetaceans broaden the known host range of *Pdd* but are currently based on limited evidence. These observations warrant targeted sampling and molecular surveillance to clarify the epidemiological role of these species. Future studies should combine microbiological, pathological, molecular, and epidemiological evidence to better define the role of *Pdd* in newly reported host species, ultimately supporting improved diagnosis and control strategies.

Current evidence consistently indicates that environmental conditions may influence both *Pdd* ecology and disease expression. Building upon these observations, this review proposes a conceptual framework in which climate change could act as a twofold driver: it may directly promote *Pdd* persistence and virulence through the upregulation of pathogenicity genes, while indirectly increasing host susceptibility by compromising immune function and ecosystem stability. Within this framework, *Pdd* could be a promising bioindicator of climate-driven shifts in coastal microbial ecology; future systematic environmental monitoring may help clarify the relationships between environmental conditions, virulence gene expression, and outbreak dynamics.

Despite the growing knowledge of *Pdd* virulence determinants, important molecular aspects of its pathogenicity remain poorly understood. In particular, little is known about the extent of molecular diversity among *Pdd* strains and the genetic basis underlying differences in host range and pathogenic potential. One particularly relevant and currently unexplored mechanistic question concerns quorum sensing (QS), a regulatory system that controls density-dependent virulence traits of bacteria. Environmental temperature is known to modulate QS networks controlling biofilm formation, adhesion, secretion system activity and other processes in several *Vibrio* species (Wang et al., 2026). Whether analogous thermal regulation of QS pathways occurs in *Pdd* remains unknown. Addressing these molecular knowledge gaps would improve our understanding of how environmental factors influence *Pdd* pathogenicity and would provide mechanistic support for the conceptual framework proposed above.

Beyond aquaculture, the ecological implications of *Pdd* remain largely unexplored. This gap is particularly relevant because *Pdd* can also persist as a free-living bacterium in marine environments, highlighting the need for an ecosystem-based approach to its study. Marine Protected Areas (MPAs) may provide valuable settings to investigate the relationships among environmental conditions, host health, and pathogen circulation, as biodiversity conservation, mitigated anthropogenic disturbance and reduced overcrowding can influence both host susceptibility and disease dynamics (Glidden et al., 2022). Long-term microbiological and molecular surveillance of both wildlife and the surrounding marine environment within MPAs could improve our understanding of the ecological drivers underlying *Pdd* persistence and disease emergence.

Similarly, rehabilitation, translocation, and reintroduction programs should consider bacterial pathogens among routine health assessments (Kock et al., 2010; Glidden et al., 2022). Because *Pdd* appears to behave as an opportunistic pathogen, physiological stress associated with capture, transport, and captivity could contribute to disease expression in subclinically infected animals. Reports of *Pdd* infection in wild-caught individuals maintained in captivity (Catanese and Grau, 2023; Bignami et al., 2026) underscore the need for systematic health screening before translocation or release.

*Pdd*’s relevance also extends beyond infection risk to food safety, as it is a recognized histamine-producing bacterium in fish and seafood products (Bjornsdottir-Butler et al., 2015; Yamaki et al., 2015), particularly under inadequate cold-chain handling. While its contribution to histamine fish poisoning remains poorly characterized, this aspect adds a potential, further dimension to the public health relevance of *Pdd* and highlights the need for integrated surveillance spanning marine environments, aquaculture, wildlife, and the seafood production chain.

### Genomic gaps and research priorities

Our analysis of publicly available genomic data reveals a significant “aquaculture bias”: the vast majority of high-quality *Pdd* genome sequences derive from farmed fish-associated isolates, while environmental, wildlife, and human-derived isolates remain markedly underrepresented (Figure 3; Supplementary Table 1). This imbalance represents a substantive limitation for the field: without genomic representation across host types and environments, it is not currently possible to identify host-adaptation signatures or to evaluate hypotheses of cross-species transmission. Therefore, sequencing environmental, human, and wildlife *Pdd* isolates, using standardized and reproducible pipelines, should be considered a research priority. Expanding genomic representation in this way would help clarify the relationship between pathogenicity, antimicrobial resistance, and environmental adaptation. Ultimately, it would contribute to a more comprehensive understanding of disease emergence as the outcome of interactions between pathogen genomic plasticity, host condition, and environmental drivers.

Integrative monitoring tools combining microbiological, genomic, climatic, and oceanographic data will be instrumental in moving from reactive surveillance toward proactive forecasting of disease emergence. Effective prevention and control of *Pdd* will therefore require interdisciplinary and ecosystem-based strategies that go beyond pathogen-specific interventions (Guardia et al., 2024). While medical treatments may mitigate impacts in aquaculture, they are insufficient in open marine environments, emphasizing the need to address underlying drivers such as anthropogenic pressures and climate change.

The transition from surveillance to mitigation will depend on coordinated international research efforts, standardized diagnostics, data-sharing, and engagement with policymakers and stakeholders. In this context, citizen science initiatives may represent a valuable complementary tool for early detection in wildlife and coastal environments. Ultimately, this review aims to support the development of targeted policies and to lay the conceptual groundwork for an integrated understanding of *Pdd* epidemiological dynamics across marine animals, wildlife, livestock, and human populations in an increasingly warming world.
