# Supplementary material for: Photobacterium damselae subsp. damselae at the animal-human interface: ecology, pathogenicity, and genomic gaps in a changing climate
Source: Front Microbiol. 2026 Aug 12;17:1901411. doi: 10.3389/fmicb.2026.1901411 (PMC13506829; doi:10.3389/fmicb.2026.1901411)
Supplement: Supplementary file 1 [file Table_1.docx]

**Table S1** Genomes included in the analysis, with NCBI accession numbers and associated metadata.

| **NCBI Accession** | **Host Organism** | **Isolation Type** | **Tissue/Organ** | **Genome Quality Metrics** |
| --- | --- | --- | --- | --- |
| **GCF_038086725.1** | Penaeus japonicus | Aquaculture | — | Complete; 4.5 Mb; 41% GC; 3 contigs; Plasmid: Yes |
| **GCF_021766015.1** | Seriola lalandi | Cell culture | Caudal kidney | Complete; 4.8 Mb; 40.5% GC; 3 contigs; Plasmid: Yes |
| **GCF_021765875.1** | Seriola lalandi | Cell culture | Caudal kidney | Complete; 4.8 Mb; 40.5% GC; 3 contigs; Plasmid: Yes |
| **GCF_021768345.1** | Seriola lalandi | Cell culture | Caudal kidney | Complete; 4.6 Mb; 41% GC; 2 contigs; Plasmid: Yes |
| **GCF_038086615.1** | Penaeus japonicus | Aquaculture | — | Complete; 4.6 Mb; 41% GC; 3 contigs; Plasmid: Yes |
| **GCF_038086915.1** | Penaeus japonicus | Aquaculture | — | Complete; 4.7 Mb; 41% GC; 5 contigs; Plasmid: Yes |
| **GCF_021768405.1** | Seriola lalandi | Cell culture | Caudal kidney | Complete; 4.6 Mb; 40.5% GC; 2 contigs; Plasmid: No |
| **GCF_021768365.1** | Seriola lalandi | Cell culture | Caudal kidney | Complete; 4.7 Mb; 40.5% GC; 2 contigs; Plasmid: No |
| **GCF_021768385.1** | Seriola lalandi | Cell culture | Caudal kidney | Complete; 4.8 Mb; 40.5% GC; 4 contigs; Plasmid: Yes |
| **GCF_021768425.1** | Seriola lalandi | Cell culture | Caudal kidney | Complete; 5.2 Mb; 40.5% GC; 3 contigs; Plasmid: Yes |
| **GCF_021768305.1** | Seriola lalandi | Cell culture | Caudal kidney | Complete; 4.8 Mb; 40.5% GC; 3 contigs; Plasmid: Yes |
| **GCF_002142615.1** | Neophocaena asiaeorientalis | Single cell isolate | Anal swab | Complete; 4.5 Mb; 41% GC; 6 contigs; Plasmid: Yes |
| **GCF_004135105.1** | Neophocaena asiaeorientalis | — | — | Complete; 4.5 Mb; 41% GC; 7 contigs; Plasmid: Yes |
| **GCF_014930975.1** | Delphinapterus leucas | — | — | Complete; 4.2 Mb; 41% GC; 3 contigs; Plasmid: Yes |
| **GCF_004794735.1** | Labrus bergylta | Cell culture | — | Contig; 4.4 Mb; 40.5% GC; 137 contigs; Plasmid: No. |
| **GCF_006297395.2** | Dicentrarchus labrax | Agar plate culture | Spleen | Contig; 4.6 Mb; 40.5% GC; 98 contigs; Plasmid: Yes. |
| **GCF_014042565.1** | Dicentrarchus labrax | Agar plate culture | Spleen | Contig; 4.8 Mb; 40.5% GC; 98 contigs; Plasmid: No. |
| **GCF_008806825.1** | Homo sapiens | Mixed culture | — | Contig; 4.4 Mb; 40.5% GC; 79 contigs; Plasmid: No. |
| **GCF_005819885.1** | Dicentrarchus labrax | Agar plate culture | Spleen | Scaffold; 4.5 Mb; 41% GC; 258 contigs; Plasmid: No. |
| **GCF_005819845.1** | Dicentrarchus labrax | Agar plate culture | Spleen | Scaffold; 4.5 Mb; 41% GC; 490 contigs; Plasmid: No. |
| **GCF_005819805.1** | Dicentrarchus labrax | Agar plate culture | Spleen | Scaffold; 4.8 Mb; 40.5% GC; 162 contigs; Plasmid: No. |
| **GCF_013377775.1** | Dicentrarchus labrax | Agar plate culture | — | Contig; 4.6 Mb; 40.5% GC; 138 contigs; Plasmid: No |
| **GCF_013377875.1** | Dicentrarchus labrax | Agar plate culture | — | Contig; 4.3 Mb; 40.5% GC; 127 contigs; Plasmid: No. |
| **GCF_005819825.1** | Dicentrarchus labrax | Agar plate culture | Kidney | Scaffold; 4.6 Mb; 40.5% GC; 157 contigs; Plasmid: No. |
| **GCF_013371545.1** | Dicentrarchus labrax | Agar plate culture | — | Contig; 4.3 Mb; 40.5% GC; 24 contigs; Plasmid: No. |
| **GCF_005819835.1** | Dicentrarchus labrax | Agar plate culture | Kidney | Scaffold; 4.8 Mb; 40.5% GC; 175 contigs; Plasmid: No. |
| **GCA_046220845.1** | Fish (aquaculture) | — | — | Contig; 4.8 Mb; 40.5% GC; 79 contigs; Plasmid: No. |
